# A Controllable and Integrated Pump-enabled Microfluidic Chip and Its Application in Droplets Generating

**DOI:** 10.1038/s41598-017-10785-1

**Published:** 2017-09-12

**Authors:** Bei Zhao, Xingye Cui, Wei Ren, Feng Xu, Ming Liu, Zuo-Guang Ye

**Affiliations:** 1 Electronic Materials Research Laboratory, Key Laboratory of the Ministry of Education & International Center for Dielectric Research, Xi’an Jiaotong University, Xi’an, 710049 China; 2 The Key Laboratory of Biomedical Information Engineering of Ministry of Education, School of Life Science and Technology, Xi’an Jiaotong University, Xi’an, 710049 China; 3 Bioinspired Engineering and Biomechanics Center (BEBC), Xi’an Jiaotong University, Xi’an, 710049 China; 4 Department of Chemistry and 4D LABS, Simon Fraser University, Burnaby, BC V54 1S6 Canada

## Abstract

A microfluidic chip with a controllable and integrated piezoelectric pump was proposed and demonstrated, where the pump was designed as a micro-actuator based on polyvinylidene fluoride (PVDF) organic piezoelectric film. In this case, the pump should integrate with the microfluidics device very well into one chip. The flow rate can be precisely controlled in the range of 0–300 µl/min for water by tuning the V_pp_ and frequency of Alternating Current (AC) voltage applied on the diaphragm. To analyze the relationship between the flow rate and the deflection of diaphragm, the deformations of diaphragm at different voltages were researched. The displacement of diaphragm was defined as 17.2 µm at the voltages of 3.5 kV, 5 Hz when the pump chamber was full of water. We have used the integrated microfluidic chip with two pumps for droplet generation to demonstrate its great potential for application in droplet-based microfluidic chip.

## Introduction

Microfluidics technology has found widespread applications in the biomedical^1–8^, chemical^9–12^, physical^13, 14^ and electronics^15–17^ areas, due to their advantageous features such as increased resolution and sensitivity, reduced sample volume, shortened turnout time and with less manual intervention^18–20^. In microfluidic chip, pumps with stable and directional fluid flow are generally required to drive flow of continuous phase or dispersed phase, where an ideal pump should be small, easy to be integrated, simple for fabrication and environment friendly to achieve well controlled, miniaturized and integrated microfluidic chip. Traditionally, two commercialized pumps contain syringe pump and peristaltic pump which are generally used in microfluidics to actuate fluid flow because of their stability, well defined directionality, wide flow rate range (minimum as 1 nl/hr. and maximum as more than one hundred microliter per minute) and low controlled error of less than ± 5%^21, 22^. Nevertheless, miniaturization and integration of microfluidic devices are difficult to be realized by these bulky pump chips, which greatly restrain their use for the microfluidic devices especially the devices that need more than one pump.

To address these issues, several self-powered or bulky facility-free pumps have been developed, such as finger or hand powered pump^23–25^ and capillary pump^26^. For instance, in finger-powered microfluidics, pushing the chamber wall by finger, the pressure of the chamber will change and then the fluid flow out of the chamber, which offers the advantages of small size, passive and low cost. However, the fluid cannot flow contiguous, the flow rate cannot be  controlled well, and more importantly it is difficult to manipulate several pumps at the same time. In capillary pump, precise fluid transport can be achieved through capillary imbibition that occurs on a programmed substrate or micro-channel, where the flow field is determined by physicochemical properties and geometrical characters of the flow domain^27^. Even though capillary pump offers the advantages of low cost, contiguous flow and easy to integrating several pumps into one chip, but it has limitations in processing large volume of fluid and modulating flow rate due to complex pump geometrical design and physicochemical modification^28^. Electromagnetic pump driven by DC motor could obtained the flow rate of ~490 µl/min under the back pressure of 592 Pa^29^. Thermal bubble actuated by nozzle-diffuser pump was used in a microfluidic mixer, the speed of mixing liquids was obtained as 6.5 µl/min. Which realized integrated the pump into the microfluidic chip successfully. But it is very difficult to control the bubble in velocity, size, position and other factors^30^.

In order to achieve precise controlled of flow in integrated microfluidic devices, many electronic materials have been used to fabricate pump, such as piezoelectric ceramics with big displacement and applied force^27, 31^, piezoelectric films integrated with silicon membrane using Micro-Electromechanical Systems (MEMS) technology^32, 33^, dielectric elastomeric with electrostriction for its flexibility and environmental performance^34^. However, piezoelectric ceramics are fragile, while dielectric elastomeric is not stable when using for a long time and the process of MEMS is complex. To address these issues, electrostrictive poly(vinylidene fluoride-trifluoroethylene) (PVDF-TrFE) thick film with a large electrostrictive strain has been used for pump, which can achieve maximum flow rate of around 25 µl/min for methanol at 63 Hz with a backpressure of 350 Pa^35^. The pump diaphragm was made up of a unimorph with the thickness of 80 µm which was formed by bonding two 20 µm thick films to an inactive PVDF polymer film using epoxy, apparently, the fabrication process is not simple enough. Besides this, the using of dilute hydrofluoric acid (HF) solution in wet etching on the glass substrate will bring about potential safety hazard and environment matter.

In this work, we developed a microfluidic chip integrated with two organic piezoelectric pumps, where the piezoelectric pump was fabricated using a thin PVDF film with the phase of β which own excellent piezoelectricity and flexibility. The PVDF film with electrodes was adhered onto the face of channel layer and served as a wall of the microchannel: this setup allows the pump element to combine with microfluidic chip consummately in order to realize a novel pump which can be integrated into one microfluidic chip with several pumps and other devices easily. The deformation of the PVDF film under electrical stimulation according to inverse piezoelectric effect will induce the volume change of the chamber and then drive the fluid flow. The PVDF piezoelectric pump can provide a wide range of flow rate (*e.g*., up to 300 µl/min for water). The directional flow was achieved by using a nozzle-diffuse design in pump chamber^36–38^. The microfluidics with droplet generating should manipulates droplets for studying rare gene expression and single-cell analysis^39, 40^. As a demonstration, we have integrated two PVDF piezoelectric pumps into one T-junction^36, 39, 40^ microfluidic chip to generate droplets, where controlled droplet size was achieved by tuning the peak to peak value of driving voltage.

## Results and Discussion

### Displacement of diaphragm

**Figure 1:**
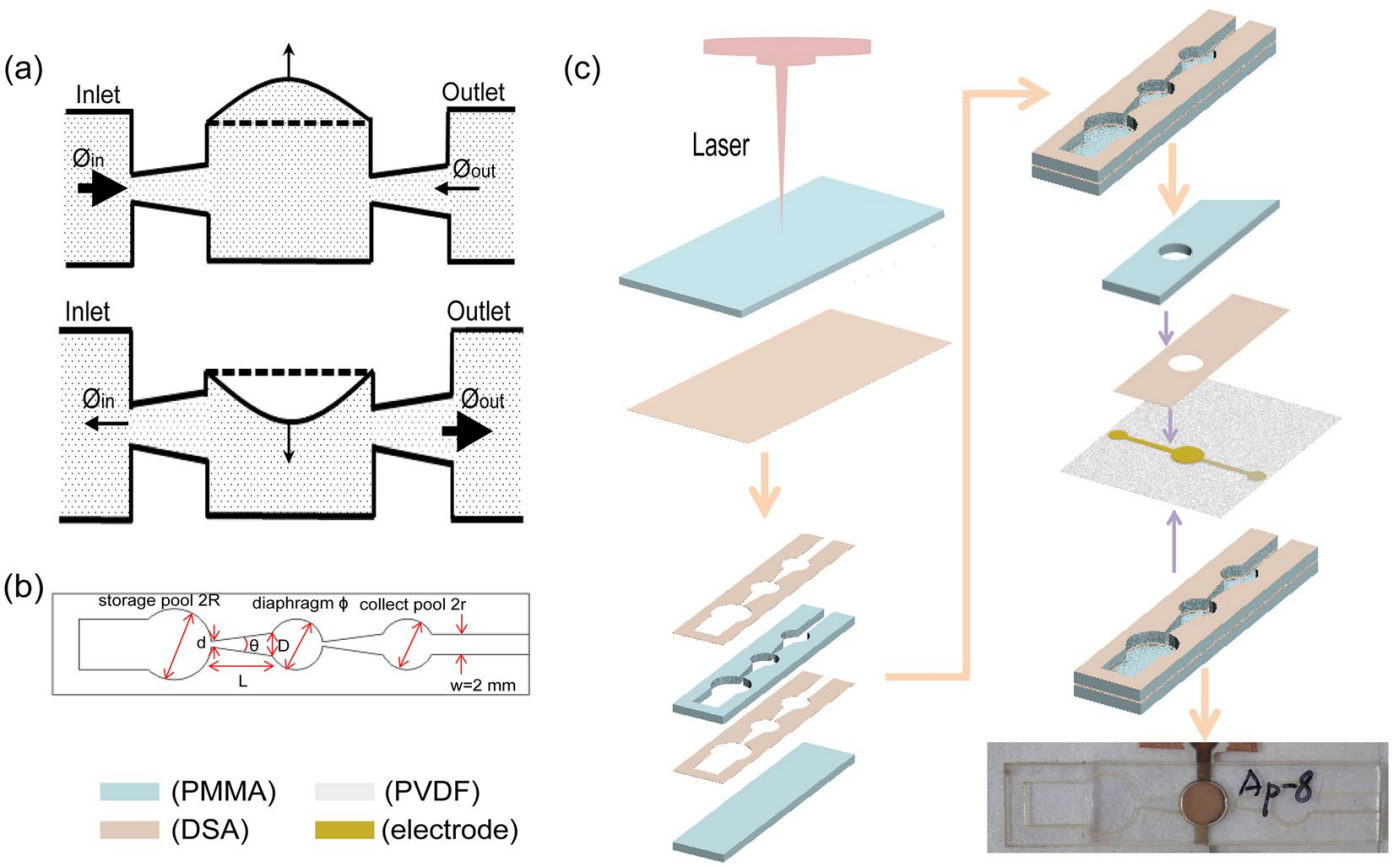
The principle of the nozzle-diffuse pump with single diaphragm, supply mode and pump mode (**a**) as the structure of the channel (**b**) fabricated by the process flow (**c**).

**Figure 2:**
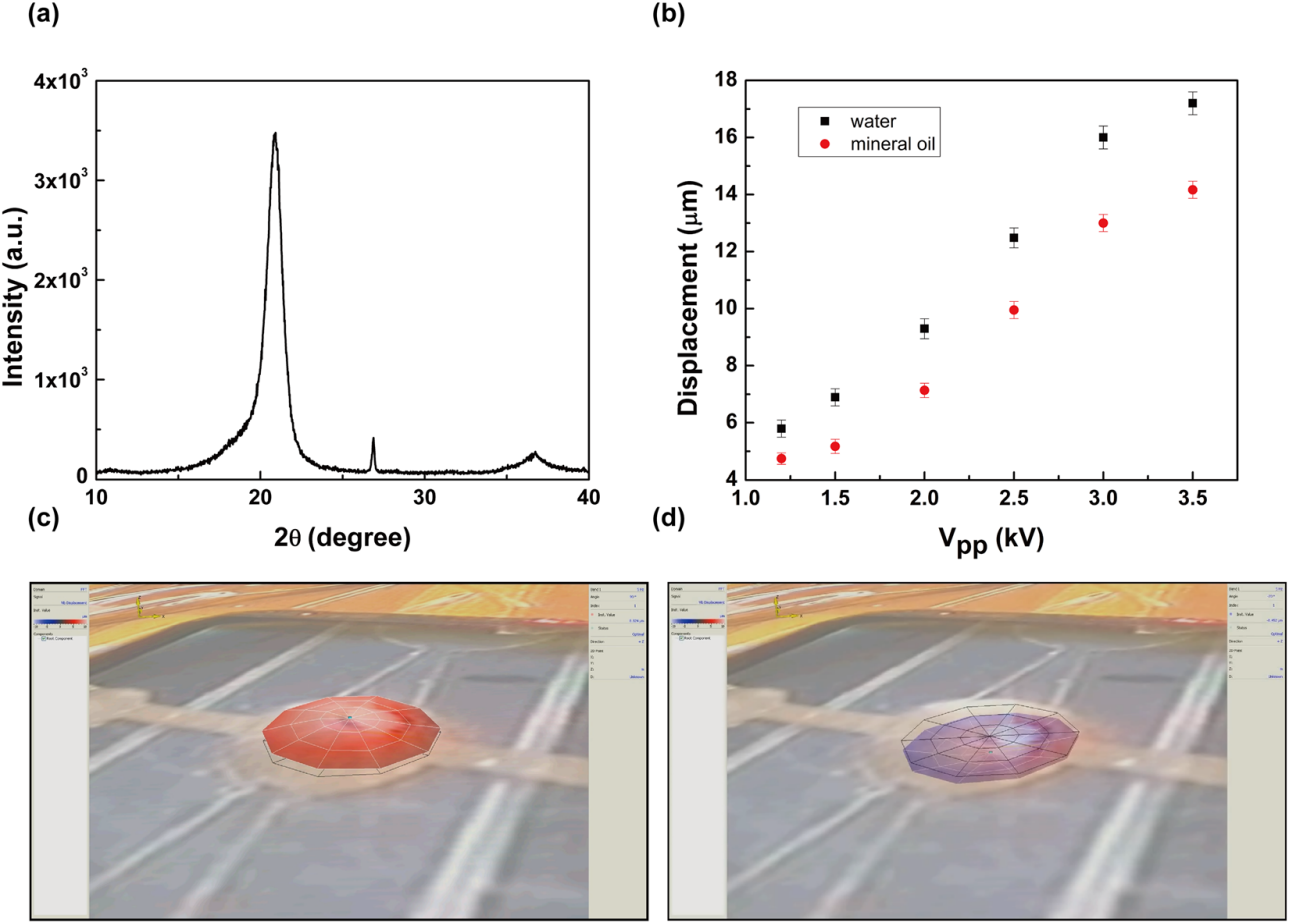
The characterization of β phase PVDF film: XRD pattern (**a**), displacement under different conditions (**b**) and the surface topography of the diaphragm when the diaphragm bend up (**c**) and down (**d**).

β phase PVDF film was used to fabricate the microfluidic pump and chip, the structure and picture of the piezoelectric pump based on PVDF are shown in Fig. 1. The crystal structure of the film was detected by x-ray diffraction (XRD), the pattern was shown in Fig. 2(a). The sharp peak at 2θ = 20.8 and 2θ = 36.7 are assigned to (110, 200) and (020, 101) reflections of PVDF crystal plane respectively which are attributed to the ferroelectric β phase. The fluid flow rate depends on the change of chamber volume which relies on the displacement of diaphragm. The performance of the pump is investigated in detail in this part. To analyze the relationship between the flow rate and the deflection of diaphragm, the deformations of the diaphragm at different voltages (V_pp_ = 1.2 kV, 1.5 kV, 2.0 kV, 2.5 kV, 3.0 kV, 3.2 kV and 3.5 kV) were measured. The animation about the diaphragm deformation at the voltage of 2 kV and the frequency of 5 Hz is shown in Supplementary Video S1. The relationships between the displacement and the voltage for different conditions are shown in Fig. 2(b), in which the chamber was full of water or mineral oil. The surface morphology of diaphragm bend to different direction, under pressure caused by increased chamber volume Fig. 2(c) the pump works as supply mode and in the other hand the pump works as pump mode as shown in Fig. 2(d). The displacement will increase with the increasing of V_pp_ when the frequency is fixed. The biggest displacement for mineral oil is 14 µm which appeared at 3.5 kV, 5 Hz which is less than for water under the same measurement conditions (17.2 µm). These results indicate that the fluids with different viscosities will experience different resistance for the oscillatory movement of diaphragm.

At low frequency, the deflection of diaphragm can reach positive maximum and then reverse as the voltage switches from ± 1/2 V_pp_ to ∓ 1/2 V_pp_. Under this condition, the film displacement will show significant change with the change of V_pp_ or frequency, where a higher V_pp_ and a lower frequency lead to a larger displacement. In comparison, at high frequency, the increased fluid loading on the diaphragm^41, 42^ deduced the displacement of diaphragm and a small change will be observed with the change of V_pp_ or frequency.

### Measurement about flow rate

**Figure 3:**
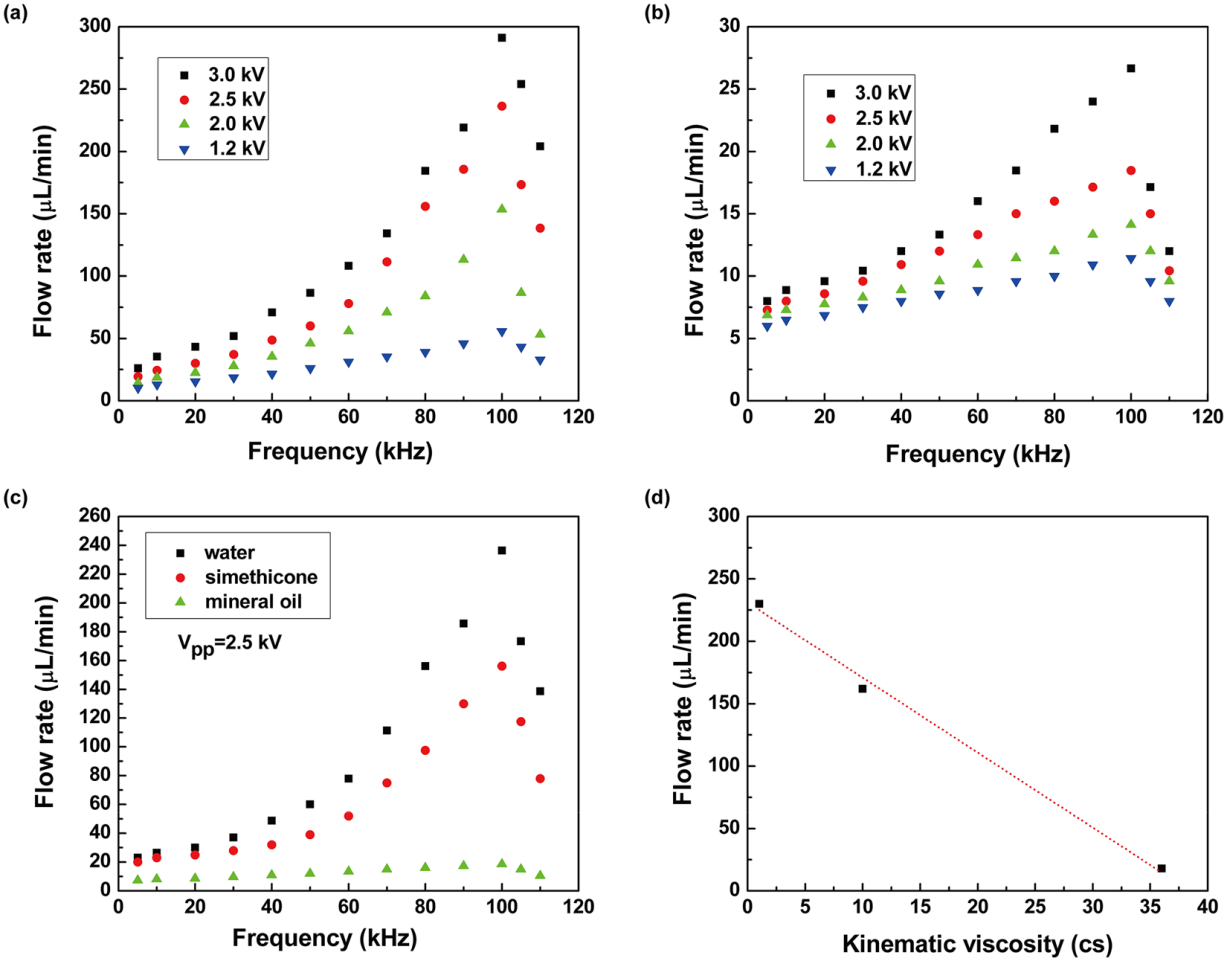
Properties of the piezoelectric pump. (**a**) relationship of fluid flow rate and frequency under different V_pp_ for water. (**b**) relationship of fluid flow rate and frequency under different V_pp_ for mineral oil. (**c**) flow rate of different fluids. (**d**) relationship of maximum fluid flow rate and kinematic viscosity.

The piezoelectric film would oscillate under the AC voltage, which will band up or down, making the volume of the chamber increase or decrease. The phenomenon of under pressure in the chamber may appear when the chamber volume increases and the pressure decreases. In this case, the liquid flows into the pump chamber, and the pump works as supply mode; on the other hand, under overpressure caused by decreased chamber volume, the liquid flows out of the pump chamber, and the pump works as pump mode (Fig. 1(a)). Based on the trapezoidal groove, the flow resistance from pump chamber to storage poll was different from pump chamber to collect poll; meanwhile, the flow resistance from storage poll to pump chamber was different from the resistance from collect poll to pump chamber. This results in the directional flow of liquid. To characterize the pump, the flow rates at different voltages for fluids with various viscosities (e.g., water (1 cs), mineral oil (36 cs) and simethicone (10 cs)) were measured and shown in Fig. 3. Under a fixed V_pp_, the fluid rate increases with increasing frequency, reaches maximum and then decreases quickly. The comparison of the flow rates for different fluids with the V_pp_ of 2.5 kV is shown in Fig. 3(c), which indicates the same tendency for three lines. The maximum flow rates show a linear relation with the viscosity, as shown in Fig. 3(d). Based on these results, we can confirm that the viscosity of the fluid is a crucial factor determining the flow rate when the V_pp_ and frequency are decided. The pump could pump water at the highest fluid rate of 300 µl/min at 100 Hz with the V_pp_ of 3 kV (as shown in Supplementary Video S2), this value is larger than those integrated pumps^23, 27^.

For a nozzle/diffuser type micro-pump, the volume flow rate can be expressed as^38^:

$${\rm{Q}}=2{\rm{\Delta }}Vf(\frac{{\eta }^{1/2}-1}{{\eta }^{1/2}+1})=2{\rm{\Delta }}Vf{\rm{C}}$$

where ∆V is the volume change of the chamber during one period of the diaphragm incentive by the alternating voltage, which is related to the displacement of diaphragm and the chamber structure, *f* is the frequency of the alternating voltage, *η* is the nozzle-diffuser efficiency which is determined by the nozzle/diffuser structure, the viscosity of fluid and the fluid flow rates in the narrow sides of the nozzle/diffusers. In the low frequency range, ∆V and *η* will almost not change with frequency, so the flow rate increasing linearly with frequency. But in the high frequency range, the ∆V decreased quickly because of the reduced displacement of diaphragm caused by the increased fluid loading on the diaphragm^41, 42^. In this case, the linearly relationship between Q and *f* is broken and Q decreasing mainly determined by the reduction of ∆V. It is obviously that, the flow rate is determined by volume change of chamber, frequency of voltage, peak to peak value of AC voltage, nozzle-diffuser structure and viscosity of fluid together. Based on the previous discussion, the volume change of chamber will decrease at high frequency, so a maximum flow rate should be obtained at one point under the resonance frequency.

For high driving voltages, a signal generator and an amplifier must be used in the testing. In order to get more portable device, an advanced high voltage source which could output high V_pp_ of AC voltage with a small size should solve this issue. Besides, we have made efforts to change the parameters of diaphragm film for the purpose of reducing the driving voltage and got some good effects.

### Droplets generating

**Figure 4:**
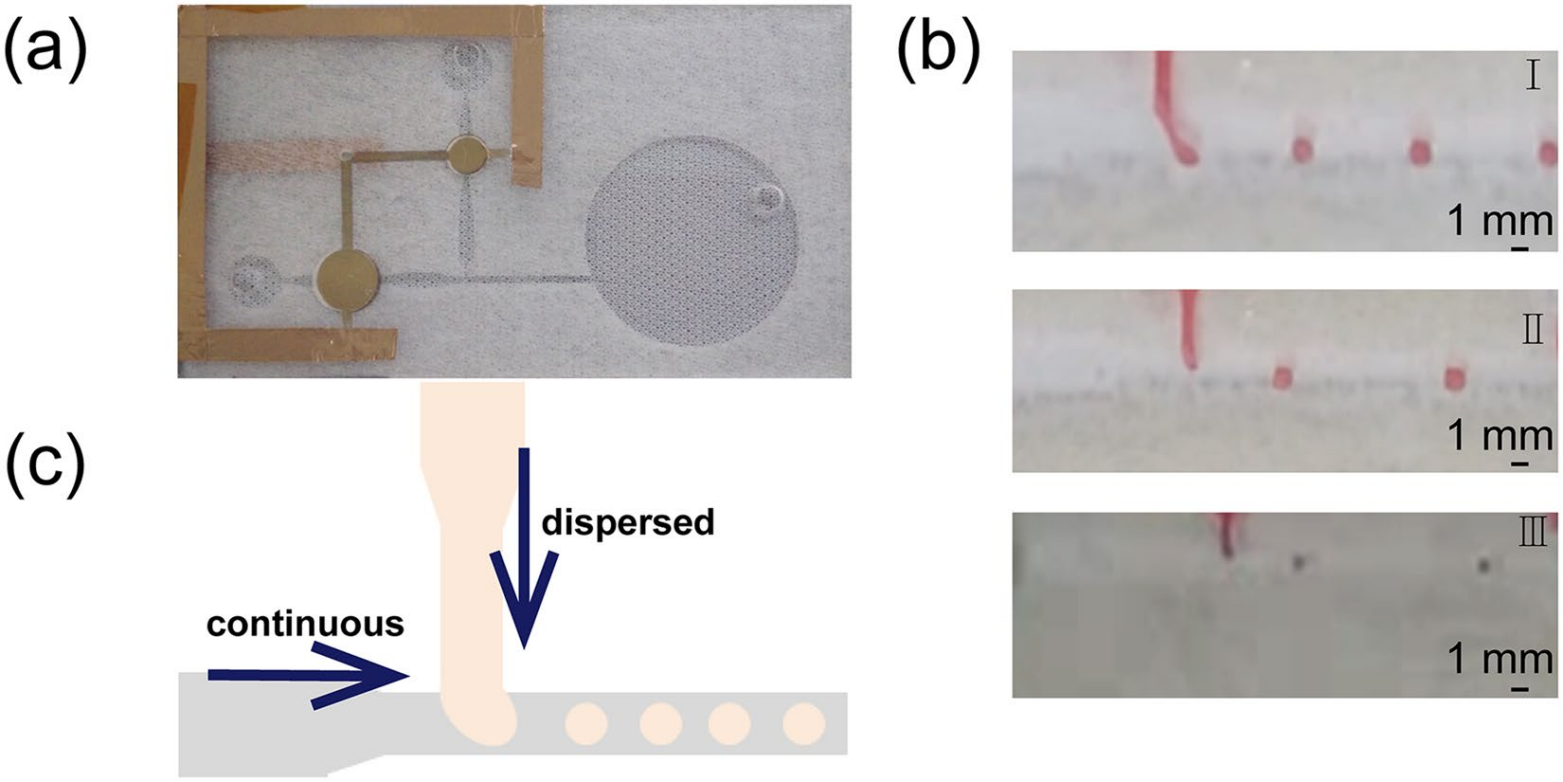
The picture (**a**) of droplet generating chip. (**b**) shows the picture of droplet generator working at 5 Hz, 1.2 kV (I); at 5 Hz, 1.5 kV (II); at 5 Hz, 2.0 kV (III). (**c**) is the schematic of T-junction.

**Figure 5:**
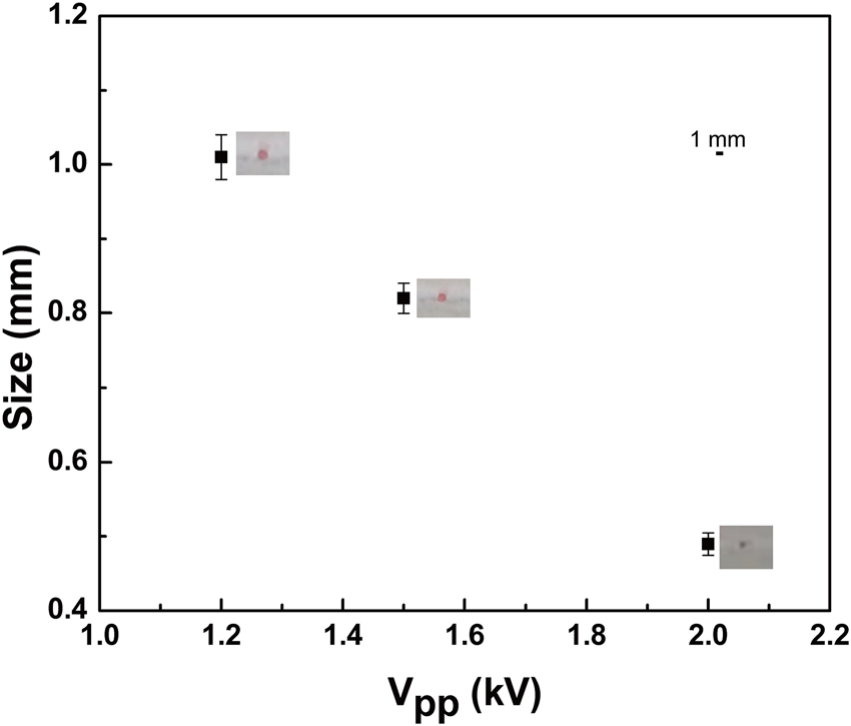
Droplet size decreased with the increasing of V_pp_.

We developed a microfluidic chip that contains several organic pumps based on a thin PVDF piezoelectric film (Fig. 4(a)). In the testing, droplets can be generated based on the T-junction structure which is shown in Fig. 4(c), the mono-dispersed water-in-oil (W/O) droplet was generated successfully and the droplets size can be controlled easily by changing the voltage. As Fig. 4(b) shows, increasing V_pp_ is equivalently to increase the flow rate at the frequency of 5 Hz, while the shear force is also increased which improve the generating rate and the distance between two neighboring droplets which are indeed a function of flow rate^43^. Different droplets sizes were obtained through adjusting the flow rate of continuous and dispersed phases by changing the V_pp_ and frequency of the applied voltage. Increasing the V_pp_ from 1.2 kV to 2.0 kV, the droplets size decreased obviously: with the diameter of 1.1 mm for 1.2 kV; 0.8 mm for 1.5 kV and 0.5 mm for 2.0 kV (Fig. 5). In the testing, the droplets could be generated stably (Supplementary Video S3, with a V_pp_ of 1.5 kV, 5 Hz), indicating that the piezoelectric film pump can serve as the drive in microfluidics system^44^.

Herein, our proposed device offers advantages in flow rate which is easy to control. Besides, its features of being soft, easy to fabricated, chemically stable, especially easy to integrate several pumps into one chip hold great potential for applications in many microfluidic fields. The pump element will also be taken into account when a microfluidic chip is designed rather than selecting one pump for a designed chip.

## Conclusions

A microfluidic chip for droplets with integrated pumps based on organic piezoelectric film was designed, fabricated and investigated. The surface topography of the diaphragm was described and the displacement of diaphragm for resonance in different chamber conditions was obtained. The flow rates of pump for different fluids were measured and the maximum flow rate was found to be linearly dependent on the viscosity of the fluid. Droplets were produced stably and the droplets size decreased with the increase of V_pp_ when the frequency was defined. It is simpler to integrate several pumps into one microfluidic chip in emerging droplet based microfluidics usage, in which cost-effective pumps integration is of great significance. This work demonstrates the feasibility of integrating delivery, mixture, detection, analysis and any other operations onto one chip with a small size and promotes the development of microfluidic chip to realize portable detection technology.

## Materials and Methods

### System design and fabrication

The microfluidic chip for droplets generating with integrated pumps based on piezoelectric film contains four layers, i.e., bottom substrate, channel layer, active layer and top substrate. The working principle and the fabrication processes of the pump and chip were shown in Fig. 1. Polymethyl methacrylate (PMMA) plate was selected to serve as channel layer and substrate layers. PVDF piezoelectric film (~28 µm, MIS, USA) was used as piezoelectric layer. These four layers were stuck together using double-sided adhesive (~50 µm, 3 M, USA).

The fluidic network was defined by top and bottom substrate layers and the channel layer, which contains pump chamber, channel, inlet port and outlet port. For the channel, there were two diffuser/nozzle elements, one for the inlet and the other for the outlet of the pump. The diffuser/nozzle element was specially designed so that the volume flowed into the diffuser direction was higher than into the nozzle direction and thus the fluid flowed from inlet to outlet under the resonance of the diaphragm. As shown in Fig. 1(b), the angle of the trapezoidal θ is 15°. The values of d, D, L and w are 1 mm, 2.6 mm, 6 mm and 2 mm, respectively. The diameters of storage poll (2R), collect poll (2r) and diaphragm (ɸ) are 7 mm, 5 mm and 5 mm, respectively.

The pattern of channel layer and substrate layer in the microfluidic system was customer-designed and then the PMMA plate was cut by a laser cutting machine (Universal VLS2.30, USA). As the first step to fabricate the pump, the channel layer was etched from a PMMA plate with a thickness of 250 µm; two pieces of double-side adhesive tape were etched as the same pattern to the channel layer and adhered onto the double faces of the channel layer. Second, the bottom and top substrate layers were cut from PMMA plate with a thickness of 500 µm. The PVDF piezoelectric film was then prepared and gold layers are deposited on the two sides of the PVDF film by a DC sputtering apparatus. Next, the bottom substrate as a complete piece of PMMA plate was pressed onto the surface of the adhesive on the bottom of the channel, the PVDF piezoelectric film with patterned electrodes was pressed onto the surface of the adhesive on the top of the channel as active layers, and the top substrate with double-side adhesive was adhered onto the top side of PVDF film (Fig. 1(c)). In the final fabrication step, silver wire with a diameter of 100 μm was adhered onto the gold electrodes by silver epoxy for electrical measurements. The total cost contains labor and material of the pump is only less than one dollar.

The picture of the chip with two pumps is shown in Fig. 4. A traditional T-junction geometry channel was used to produce droplets, there are two pumps in this microfluidic chip, one transport the continuous phase and the other transport the dispersed phase and the dispersed phase perpendicularly intersects the continuous phase. The shrink of the dispersed phase was 0.5 mm, the width of the continuous phase channel was 1 mm, and the diameter of the pump is 8 mm for continuous phase and 5 mm for dispersed phase. Due to the shear force from the continuous phase, the dispersed phase was broken up so the droplets were generated at the cross of the two phases. Furthermore, multiple channels chip has also been designed (Supplementary Figure S1).

### Surface morphology

In order to describe the oscillatory movement of piezoelectric film, a scanning Laser Doppler Vibrometer (PSV-400, PolyTec, Germany) was used as a typical non-contact measuring instrument. This equipment contains many advantages like no added mass effect, strong anti-interference ability, field application, convenient measurement and high precision and so forth, so it is very suitable for explosion and impact test. This diaphragm morphology is visualized by reflection digital holography microscopy: the advantage is its capability of fast detection of the diaphragm resonating under different voltage without perturbing the device operation. The laser beam is focused using the focusing lens and split into two parts by the beam splitter. The microscopic objective is placed at one side of the beam splitter and the focusing lens is adjusted such that the object beam becomes collimated. The other beam is focused on a plane mirror and reflects back. The object beam and the reference beam are reflected by the mirror, and after reflection, interfere and are recorded by the CCD.

The helium-neon laser beam was directed to the resonating diaphragm and the scattered light was collected and interfered with the reference beam on a photodetector. The output of the photodetector was a standard frequency spectrum representing the velocity or displacement of the diaphragm. The diaphragm was excited by applying a periodic chirp signal on the pump, so the surface morphology under different conditions of the voltage should be obtained. The excitation voltage (square wave) was changed from 1.2 kV to 3.5 kV, and the frequency was increased from 5 Hz to 110 Hz.

### Characterization

A square wave excitation signal was used to drive the pump which was supplied by a signal generator (ROGOL, China) and an amplifier (610E, Trek, USA). For the convenience of observation, food coloring solution was chosen as the test fluid to detect the fluid flow rate. The experimental process was as follows: (i) drop a solution of 20 µL into the storage pool to fill it up; (ii) turn on the power and start the time; (iii) when the storage pool is empty, stop the power and the time, and (iv) calculate the fluid flow rate from the elapsed time and the volume of the storage pool. The flow rates for water (food coloring solution) at different voltage parameters were obtained through changing the frequency and peak-to-peak value (V_pp_) of AC voltage. The flow rates for mineral oil (M5310, Sigma Aldrich) with a viscosity of about 36 cs and simethicone with a viscosity of 10 cs could also be obtained using the same method. The stability of the pump can be described through measuring the flow rate when it was working continuously. All of the tests were carried out at room temperature.

In the test of droplet creating, mineral oil was used as the continuous phase and food coloring solution was used as the dispersed phase. Mono-dispersed water-in-oil (W/O) droplet was generated due to the shear force.

## Electronic supplementary material

Supplementary information

supplementary video 1

supplementary video 2

supplementary video 3

## References

[1] Bithi SS, Vanapalli SA (2017). Microfluidic cell isolation technology for drug testing of single tumor cells and their clusters. Sci Rep.

[2] Kim S (2017). High-throughput automated microfluidic sample preparation for accurate microbial genomics. Nat Commun.

[3] Jiang H (2017). Integrated Microfluidic Flow-Through Microbial Fuel Cells. Sci Rep.

[4] Beebe DJ, Mensing GA, Walker GM (2002). Physics and applications of microfluidics in biology. Annu Rev Biomed Eng.

[5] Tokel O (2015). Portable microfluidic integrated plasmonic platform for pathogen detection. Sci Rep.

[6] Sackmann EK, Fulton AL, Beebe DJ (2014). The present and future role of microfluidics in biomedical research. Nature.

[7] Wang S (2013). Point-of-care assays for tuberculosis: role of nanotechnology/microfluidics. Biotechnol Adv.

[8] Lee WG (2010). Nano/Microfluidics for diagnosis of infectious diseases in developing countries. Adv Drug Deliv Rev.

[9] Jiang J (2017). An integrated microfluidic device for rapid and high-sensitivity analysis of circulating tumor cells. Sci Rep.

[10] Valencia PM, Farokhzad OC, Karnik R, Langer R (2012). Microfluidic technologies for accelerating the clinical translation of nanoparticles. Nat Nanotechnol.

[11] Mark D (2010). Microfluidic lab-on-a-chip platforms: requirements, characteristics and applications. Chem Soc Rev.

[12] Elvira KS, Casadevall i Solvas X, Wootton RC, deMello AJ (2013). The past, present and potential for microfluidic reactor technology in chemical synthesis. Nat Chem.

[13] Monat C, Domachuk P, Eggleton BJ (2007). Integrated optofluidics: A new river of light. Nat Photon.

[14] Bayraktar T, Pidugu SB (2006). Characterization of liquid flows in microfluidic systems. International Journal of Heat and Mass Transfer.

[15] Xu S (2014). Soft Microfluidic Assemblies of Sensors, Circuits, and Radios for the Skin. SCIENCE.

[16] Persano L (2013). High performance piezoelectric devices based on aligned arrays of nanofibers of poly(vinylidenefluoride-co-trifluoroethylene). Nat Commun.

[17] Groisman A, Enzelberger M, Quake SR (2003). Microfluidic Memory and Control Devices. Science.

[18] Whitesides GM (2006). The origins and the future of microfluidics. Nature.

[19] Whitesides GM (2013). Cool, or simple and cheap? Why not both?. Lab Chip.

[20] Hu J (2014). Advances in paper-based point-of-care diagnostics. Biosens Bioelectron.

[21] Li Z, Mak SY, Sauret A, C HC (2014). Syringe-pump-induced fluctuation in all-aqueous microfluidic system implications for flow rate accuracy. Lab Chip.

[22] Yobas L, Tang KC, Yong SE, Kye-Zheng Ong E (2008). A disposable planar peristaltic pump for lab-on-a-chip. Lab Chip.

[23] Gong MM, Macdonald BD, Vu Nguyen T, Sinton D (2012). Hand-powered microfluidics: A membrane pump with a patient-to-chip syringe interface. Biomicrofluidics.

[24] Holmes DP, Tavakol B, Froehlicher G, Stone HA (2013). Control and manipulation of microfluidic flow via elastic deformations. Soft Matter.

[25] Elizalde E, Urteaga R, Berli CLA (2015). Rational design of capillary-driven flows for paper-based microfluidics. LAB ON A CHIP.

[26] Kokalj T (2014). Self-powered imbibing microfluidic pump by liquid encapsulation: SIMPLE. Lab on a Chip - Miniaturisation for Chemistry and Biology.

[27] Ren K, Chen Y, Wu H (2014). New materials for microfluidics in biology. Curr Opin Biotechnol.

[28] Iwai K (2014). Finger-powered microfluidic systems using multilayer soft lithography and injection molding processes. Lab Chip.

[29] Du M, Ye X, Wu K, Zhou Z (2009). A peristaltic micro pump driven by a rotating motor with magnetically attracted steel balls. Sensors (Basel).

[30] Tsai J-H, Lin L (2002). Active microfluidic mixer and gas bubble filter driven by thermal bubble micropump. Sensors & Actuators: A. Physical.

[31] Friend, J. & Yeo, L. Piezoelectric Materials for Microfluidics, *Springer - Book Chapter* (2008).

[32] Ham Y-B (2010). Development of a piezoelectric pump for a highly-precise constant flow rate. Journal of the Korean Physical Society.

[33] Leng X-F (2013). Theory and experimental verification of spiral flow tube-type valveless piezoelectric pump with gyroscopic effect. Sensors and Actuators, A: Physical.

[34] Tavakol B (2014). Buckling of dielectric elastomeric plates for soft, electrically active microfluidic pumps. Soft Matter.

[35] Xia F, Tadigadapa S, Zhang QM (2006). Electroactive polymer based microfluidic pump. Sensors & Actuators: A. Physical.

[36] Anna SL, Bontoux N, Stone HA (2003). Formation of dispersions using “flow focusing” in microchannels. Applied Physics Letters.

[37] Olsson, A., Enoksson, P., Stemme, G. & Stemme, E. An improved valve-less pump fabricated using deep reactive ion etching. *Proceedings of Ninth International Workshop on Micro Electromechanical Systems* 479 (1996).

[38] Olsson A, Stemme G, Stemme E (1995). A valve-less planar fluid pump with two pump chambers. Sensors and Actuators: A. Physical.

[39] Dangla R, Kayi SC, Baroud CN (2013). Droplet microfluidics driven by gradients of confinement. Proc Natl Acad Sci USA.

[40] Teh SY, Lin R, Hung LH, Lee AP (2008). Droplet microfluidics. Lab Chip.

[41] Xu TB, Cheng ZY, Zhang QM (2002). High-performance micromachined unimorph actuators based on electrostrictive poly(vinylidene fluoride–trifluoroethylene) copolymer. Applied Physics Letters.

[42] Olsson A, Stemme G, Stemme E (1999). A numerical design study of the valveless diffuser pump using a lumped-mass model. Journal of Micromechanics and Microengineering.

[43] Zhang, Y. & Liu, H. Droplet formation in microfluidic cross-junctions. *Physics of Fluids* **23**, 082101–082112 (2011).

[44] Harris, D. M., Liu, T. & Bush, J. W. M. A low-cost, precise piezoelectric droplet-on-demand generator. *Experiments in Fluids* **56** (2015).

