# Supplementary material for: A Controllable and Integrated Pump-enabled Microfluidic Chip and Its Application in Droplets Generating
Source: Sci Rep. 2017 Sep 12;7:11319. doi: 10.1038/s41598-017-10785-1 (PMC5596006; doi:10.1038/s41598-017-10785-1)

A Controllable and Integrated Pump-enabled Microfluidic Chip and Its Application in Droplets Generating

Bei Zhao[1,+], Xingye Cui[2,3,+], Wei Ren[1,*], Feng Xu[2,3,*], Ming Liu[1], Zuo-Guang Ye[4,1]

[1] Electronic Materials Research Laboratory, Key Laboratory of the Ministry of Education & International Center for Dielectric Research, Xi'an Jiaotong University, Xi'an, 710049, China

[2] The Key Laboratory of Biomedical Information Engineering of Ministry of Education, School of Life Science and Technology, Xi'an Jiaotong University, Xi'an 710049, China

[3] Bioinspired Engineering and Biomechanics Center (BEBC), Xi'an Jiaotong University, Xi'an 710049, China

[4] Department of Chemistry and 4D LABS, Simon Fraser University, Burnaby, BC, V54 1S6, Canada

[+]These authors contribute equally to this work.

[*] The corresponding author. Corresponding E-mail, wren@mail.xjtu.edu.cn; fengxu@mail.xjtu.edu.cn

Supplementary information:

Video S1 shows the animation about the diaphragm deformation with the V_{pp} of 2 kV and the frequency of 5 Hz;

Video S2 shows the pump is working at the excitation voltage of 3 kV (V_{pp}), 110 Hz (frequency);

Video S3 shows the droplet generating chip is working at the excitation voltage of 1.5 kV (V_{pp}), 5 Hz (frequency);

Figure S1 shows the droplet generating chip with multiple channels by integrating four pumps together.

Figure S1:

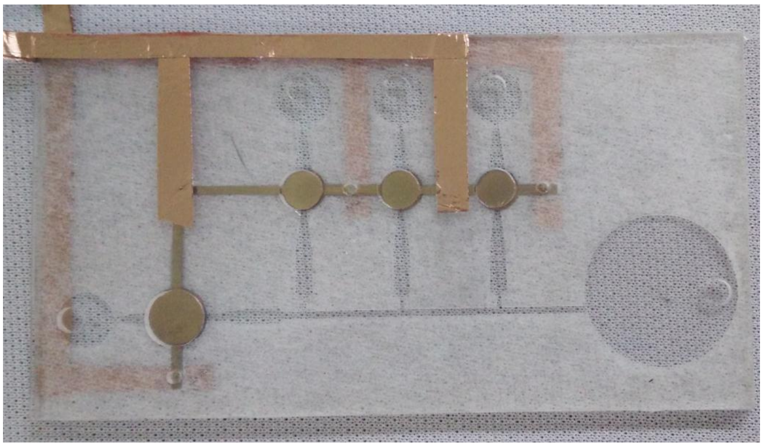

Supplement: Supplementary file 1 — Supplementary information [file 41598_2017_10785_MOESM1_ESM.pdf]
